# Illness Perceptions, Coping, Health-Related Quality of Life and Psychological Outcomes in Cervical Dystonia

**DOI:** 10.1007/s10880-022-09851-2

**Published:** 2022-04-19

**Authors:** Sarah O’Connor, David Hevey, Fiadhnait O’Keeffe

**Affiliations:** 1 School of Psychology, Trinity College Dublin, Dublin, Ireland; 2 Department of Psychology, St Vincent’s University Hospital, Dublin, Ireland

**Keywords:** Cervical dystonia, Illness perceptions, Coping, Quality of life, Anxiety, Depression

## Abstract

This study examined the predictive ability of the Common-Sense Model to explain psychological outcomes in cervical dystonia, a movement disorder that affects the muscles of the head and neck. Illness Perceptions Questionnaire-Revised, brief COPE, Hospital Anxiety and Depression Scale, Cervical Dystonia Impact Profile and the Post-Traumatic Growth Inventory were completed by 118 people with cervical dystonia. Correlations and hierarchical multiple regression models were conducted. Illness perceptions and coping strategies explained 59% of variance in anxiety, 61% of variance in depression and health-related quality of life and 19% of variance in post-traumatic growth. Illness perceptions and coping strategies are significant factors to consider in terms of psychological adjustment and outcomes in cervical dystonia. Psychological interventions targeting illness perceptions and coping strategies may be beneficial in improving psychological outcomes for people with cervical dystonia.

**Supplementary Information:**

The online version contains supplementary material available at 10.1007/s10880-022-09851-2.

## Illness Perceptions, Coping and Psychological Outcomes in Cervical Dystonia

Dystonia is a movement disorder, characterised by sustained or intermittent muscle contractions that cause repetitive movements and postures (Albanese et al., 2013). It is the third most common movement disorder after Parkinson’s disease (PD) and essential tremor (Defazio, 2010). Cervical dystonia (CD) is the most common form of adult onset isolated dystonia (AOIFD), with an estimated prevalence rate of 16.43 per 100,000 (Steeves et al., 2012).

CD is characterised by uncontrollable twisting and turning of the head due to painful involuntary contractions of the neck muscles. Treatment for CD is typically focused on alleviating motor severity. Botulinum toxin (BTX) injections into the effected muscles every 12–16 weeks are the primary and most effective form of treatment (Albanese et al., 2019).

The conceptualisation of dystonia as solely a movement disorder is being challenged by the growing evidence that suggests a constellation of non-motor symptoms. Pain, sensory abnormalities, sleep, and psychological difficulties are frequently reported and are often present months or years before motor symptom onset (Smit et al., 2017; Stamelou et al., 2012). These non-motor difficulties have been shown to have a greater impact on health-related quality of life (HRQoL), above and beyond the impact of motor symptoms (Ndukwe et al., 2020a, 2020b). CD is associated with increasing functional impairments, including reduced working ability (De Pauw et al., 2017; Ortiz et al., 2019. Up to 71% of people with dystonia met the criteria for a diagnosis of anxiety or depression over the course of their lifetime (Kuyper et al., 2011). The high levels of distress have led to debates as to whether depression and anxiety are a primary feature or a secondary consequence of living with dystonia. Some studies have shown that depression and anxiety often precede the onset of dystonia, while others have shown that psychosocial variables such as self-esteem, body image, social isolation, stigma and coping have a significant impact on mood and quality of life (Comella & Bhatia, 2015; Lewis et al., 2008; Morgan et al., 2019; Ndukwe et al., 2020a, 2020b). The diagnosis of a chronic health condition requires adjustment across multiple life domains and is frequently linked to increased distress (Stanton et al., 2007). Like other movement disorders, a biopsychosocial framework as an alternative to the medicalised understanding of distress is recommended (British Psychological Society [BPS], 2021; Wade & Halligan, 2017).

The common-sense model (CSM; Leventhal et al., 2003) is a sociocognitive model that has been widely applied to other chronic physical health conditions to explain psychological outcomes such as depression, anxiety and HRQoL. According to the CSM, when faced with a health threat, an individual develops a set of cognitive and emotional representations of the illness. These representations determine what cognitive and behavioural coping strategies are selected to manage the impact of the health threat, which are then appraised in terms of their efficacy for managing the threat (Leventhal et al., 2016). Through understanding the relationships between perceptions, coping and outcomes, it has been suggested that tailored psychological interventions can be developed (Graham et al., 2013; Wearden & Peters, 2008).

Illness perceptions are shaped by an individual’s sociocultural background, previous experiences, and availability of lay or expert information (Hagger et al., 2017). There are several distinct and inter-related illness perceptions; identity (the symptoms and labels associated with an illness), consequences (the impact of the illness on functioning), timeline (the duration of the illness as chronic or acute/cyclical), control (how much personal control or treatment control influences the illness), coherence (how much an individual understands and makes sense of their illness), emotional representations (the emotional impact or response to the illness) and causes (possible attributions). While illness perceptions have not been formally examined in people with cervical dystonia (pwCD), Comella & Bhatia (2015) undertook an international survey (*N* = 1071) to assess pwCD beliefs around the impact of CD and expectations for treatment. CD was reported to have a significant impact on well-being, health and work. Pain and mood difficulties were frequently reported and expectations for treatment were high; over half expected to be able to return to a normal routine and over 60% expected to be free from pain and spasms following treatment (Comella & Bhatia, 2015).

Within the CSM and wider literature, coping strategies are typically conceptualised as avoidant or emotion focused and active or problem focused (Hagger & Orbell, 2021; Stanton et al., 2007). Recent meta-analyses on a range of health conditions have shown that threat-based perceptions, such as identity, consequences, timeline and emotion representations are related to greater avoidant or emotion-focused coping strategies and subsequently to increased distress and lower quality of life, whereas coherent and control-related beliefs have been associated with the use of active and problem-focused coping and subsequently with more positive outcomes (Dempster et al., 2015; Hagger & Orbell, 2021).

While the CSM and related illness perceptions have not yet been studied in CD, coping and psychological outcomes have been examined in three studies. Jahanshahi (1991) found that maladaptive coping strategies such as wish-fulfilling fantasy and religious faith explained 1.7% of variance in depression scores in the study of pwCD. Depressive coping, which included blaming oneself, feeling guilty and wishful thinking, was related to anxiety and depression by Scheidt et al. (1998), and was also found to be a significant predictor of depression, anxiety and social phobia in Gündel et al. (2003).

There is often a tendency to focus on measuring distress and other indicators of poor adjustment in chronic illnesses (Barak & Achiron, 2009; Stanton et al., 2007). In other chronic conditions, perceived personal growth has been described as the process of positive change that develops as a result of challenges and adversity associated with living with a chronic condition (Tedeschi & Calhoun, 1996) and has been demonstrated to have a positive impact on physical and mental health outcomes (Zeligman et al., 2018). Positive changes or growth have been found in other chronic neurological conditions including acquired brain injury and multiple sclerosis (MS) (Ackroyd et al., 2011; Rogan et al., 2013). Post-traumatic growth (PTG) has also been examined in blepharospasm (BSP), another form of AOIFD (Nikolai et al., 2016). Relative to a hemi-facial spasm clinical control group, people with BSP were found to have significantly higher levels of post-traumatic growth. The authors attributed these higher growth scores to people with BSP perceiving their illness as more debilitating and socially devastating (Nikolai et al., 2016). To date, positive changes and growth have not been examined in pwCD.

The CSM has proved a valuable psychological framework to study and understand psychological outcomes in a variety of chronic neurological and physical health conditions including MS, brain injury, muscle disorders, inflammatory bowel disease and alopecia (Bassi et al., 2020; Cartwright et al., 2009; Graham et al., 2013; Snell et al., 2011; van Erp et al., 2017); however, to date, it has not been applied to pwCD. The current study applies the CSM in pwCD to examine its explanatory ability. Specifically, this study aims to explore the relationships between illness perceptions and coping strategies for pwCD, in addition to exploring to what extent illness perceptions and coping explain psychological outcomes in pwCD. In line with the assumptions of the CSM, it was hypothesised that: (1) negative illness perceptions would be associated with higher levels of distress and lower HRQoL, (2) avoidance-focused coping would be related to higher distress and lower HRQoL, (3) problem-focused coping would be related to lower distress and better HRQoL, and (4) illness perceptions and coping strategies will explain a significant amount of variance in psychological outcomes and HRQoL. The current study also examined whether pwCD experienced a growth in their lives and a measure of PTG was included as an additional outcome of psychological well-being.

## Method

### Procedure and Participants

This study was cross-sectional in design. Ethical approval was granted from both a University teaching hospital (Ref: RS20-009) and University (Ref: SPREC062020-05). Inclusion criteria were adults over the age of 18, with a diagnosis of cervical dystonia, and sufficient understanding of English to complete the questionnaires. Individuals were excluded if they indicated having another form of dystonia (e.g. generalised or other focal dystonia), if their dystonia was secondary to an illness or injury or if they reported any other history of developmental or neurological illness.

The survey pack was posted to 198 pwCD, attending a University teaching hospital for treatment in September 2020, who had previously consented to be contacted about future research. The survey was also advertised online through a dystonia charity. Recruitment was closed in February 2021 and no incentives were used. Of the 198 pwCD who were eligible, 115 pwCD returned the questionnaire pack, giving a participation rate of 58.1%. Forty-four pwCD opened the online version of the survey and 18 complete datasets were obtained, a 40.9% response rate.

### Measures

#### Demographics

Self-reported demographic data were collected on a specifically designed questionnaire for the study. Age, age of CD onset, treatment, medical history, and lifestyle history were recorded.

#### Illness Perceptions

Participants’ illness perceptions were measured using the Illness Perceptions Questionnaire- Revised (IPQ-R, Moss-Morris et al., 2002). The IPQ-R assesses different components of illness perceptions: identity, beliefs and causes. The identity subscale provides a list of different illness symptoms: participants report whether they experienced this symptom and if it was related to their CD. The second part of the questionnaire includes 38 items to assess participants’ beliefs about CD on a scale of 1 (strongly disagree) to 5 (strongly agree); timeline acute/chronic or cyclical, personal control, treatment control, illness coherence and emotional representations. The third part assesses beliefs about the causes of CD, rated from 1 (strongly disagree) to 5 (strongly agree). Three causal attributions, explaining 49.24% of the variance were extracted using principal component analysis (PCA): psychosocial causes, external/immunity and risk factor behaviours. Although not previously used in pwCD, it has been used in other neurological such as MS (Bassi et al., 2020). The IPQ-R has demonstrated good internal reliability and validity (Moss-Morris et al., 2002). Internal consistencies of the subscales were good in the current study, ranging from *α* = 0.63–0.90, please see Table 2.

#### Coping

The Brief COPE (Carver, 1997) measures 14 different coping strategies, with higher scores indicating more frequent use of that strategy. Individuals indicated how much they had been using each strategy to deal with stress since their CD diagnosis. Items were scored on a 4-point scale from 1 (I have not been doing this at all) to 4 (I have been doing this a lot). Using PCA analysis, five higher-order coping strategies, explaining 58.51% of the variance were extracted. The first factor included active coping, planning and support items was labelled problem-focused coping (*n* = 9), explained 26.30% of the variance. The second factor, comprising items such as denial and behavioural disengagement (*n* = 6), explained 11.62% of the variance. Spirituality (*n* = 2), humour (*n* = 2) and substance use (*n* = 2), all comprised of their respective items explained 7.66%, 6.85% and 6.06% of variance, respectively, Please see Supplementary Information for further details. The Brief COPE has demonstrated good psychometrics in other neurological conditions, including MS (Bassi et al., 2020) and acquired brain injury (Rogan et al., 2013). It is commonly used in studies applying the CSM (Hagger et al., 2017). Internal consistencies of the subscales in the current study were good, ranging from *α* = 0.76–0.89.

#### Psychological Distress

The Hospital Anxiety and Depression Scale (Zigmond & Snaith, 1983) is a 14-item self-report measure of depression (HADS-D) and anxiety (HADS-A). Items are rated on a 0–3-point scale, and each subscale has a range from 0 to 21, with higher scores indicating greater distress. The presence of anxiety or depression was determined based on the cut-off of > 8 for mild and > 11 for clinical levels of distress (Bjelland et al., 2002). The HADS is widely used in physical health conditions including CD (Berman et al., 2017) and has demonstrated good validity and consistency in physical health conditions (Bjelland et al., 2002). Internal consistency was excellent in the current study (*α* = 0.90).

#### Health-Related Quality of Life

The Cervical Dystonia Impact Profile-58 (CDIP-58) is a 58-item scale that provides a measure of the impact of CD across eight HRQoL domains: head and neck symptoms, pain and discomfort, upper limb activity, annoyance, sleep, lower limb activity, mood and psychosocial functioning (Cano et al., 2004). The total score was used as an outcome variable, with higher scores indicating poorer HRQoL. The CDIP-58 has demonstrated excellent validity and reliability (Cano et al., 2008). Internal consistency in the current study was excellent (*α* = 0.98).

#### Post-Traumatic Growth

The Post-Traumatic Growth Inventory (PTGI) is a 21-item measure of positive changes or growth. Each item is rated on a six-point scale from 0 (I did not experience this change) to 5 (I experienced this change to a very great degree). It has demonstrated good validity and reliability (Tedeschi & Calhoun, 1996). The total score was used as an outcome variable in the current study. The PTGI has not been used in CD, although it has been used in BSP (Nikolai et al., 2016). Internal consistency was excellent in the current study (Cronbach’s alpha = 0.96).

### Statistical Analysis

A priori power analysis using G*Power (Faul et al., 2009) indicated that a sample of 123 provided power of .80 to detect a medium effect size (*f*^2^ = 0.15) in multiple regression with eleven independent predictors at *p* < .05. One hundred and thirty-three questionnaires were received. Data were analysed using SPSS version 26.0 (IBM Corp, 2019). Missing values analysis was conducted, and 6 cases missing multiple data points from multiple measures were removed (missing > 2 data points per measure). Nine other participants did not complete full scales required for the analysis (CDIP-58, *n* = 3; COPE, *n* = 2; PTGI, *n* = 4) and were also removed, leaving *n* = 118 for the final analysis**.** Principal component analysis with varimax rotation was used for the cause subscale of the IPQ-R and the Brief COPE, guided by Kaiser’s criterion (eigenvalues > 1), scree plot inspection and supported by Parallel Analyses. Items were retained if they showed loadings of > .50 on one factor and < .40 on another factor (Bassi et al., 2020). Descriptive statistics and internal reliabilities for each scale were calculated. For preliminary analyses, data were screened for normality, skewness and kurtosis, and nonparametric correlations were used to examine the main study variables. Statistical significance for initial exploratory analyses was set at *p* < .05. Variables that met statistical significance at *p* < .01 were entered in the regression models. After checking the relevant assumptions of multivariate normality, linearity, multicollinearity and homoscedasticity, four separate hierarchical regressions were carried out. Potential confounders and significant demographics were entered in block 1, illness perceptions in block 2 and coping strategies in block 3.

## Results

### Demographics

Sociodemographic details are presented in Table 1. Participants were mostly female (73.7%) and living with a partner, family or friends (78.8%). The mean age of the sample was 59 (*SD* = 13.38) and average disease duration was 14.55 years (*SD* = 10.85). Most of the sample reported receiving botulinum toxin A (BTX) injections (94.1%) and over half were using other treatments to manage their symptoms (42.3% other medications and 12.8% other treatments such as physiotherapy).

**Table 1:** Sociodemographic details (*N* = 118)

	*n*	%
Gender		
Female	87	73.7
Male	31	26.3
Botox injections	111	94.1
Medications	50	42.4
Other treatment	15	12.7
Physical health comorbidities	7	5.9
History of mental health difficulties	32	27.1
Education (*n* = 117)		
Primary	10	8.5
Second level	51	43.2
Third level UG	25	21.2
Third level PG	21	17.8
Professional training	7	5.9
Apprenticeship/other	3	2.6
Employment		
Not working	21	17.8
Working full time	30	25.4
Working part time	17	14.4
Retired	45	38.1
Other	5	4.2
Relationship status (*n* = 117)		
Single	18	15.3
Married/partner	74	62.7
Widowed	13	11
Other	12	10.2
Living arrangements (*n* = 117)		
Alone	21	17.8
With others	93	78.8
Other	3	2.6

### Psychological Characteristics

Twenty-seven per cent reported a previous history of mental health difficulties. According to the HADS, 15.3% (*n* = 18) presented with mild anxiety (> 8), while 35.6% (*n* = 42) presented with clinically significant anxiety (> 11). Mild depression was evident in 10.2% (*n* = 12), while 18.6% (*n* = 22) presented with clinically significant depression. The mean score for the CDIP-58 was 35.83 (*SD* = 23.98), which is low relative to the scale range, which indicates a higher HRQoL. Mean score for the PTGI was 33.62 (*SD* = 25.45), which is similarly low relative to the scale range, indicating low levels of PTG. Please see Table 2.

**Table 2:** Descriptive statistics and internal reliability of study variables

	Mean (SD)	Range	Possible range	*α*
Outcomes				
HADS-A	8.26 (5.65)	0–19	0–21	.90
HADS-D	5.68 (4.92)	0–19	0–21	.90
CDIP-58	35.83 (23.98)	0–92	0–100	.98
PTGI	33.62 (25.45)	0–105	0–105	.96
Illness perceptions				
Identity	3.57 (3.23)	0–13	0–13	–
Timeline (chronic)	26.00 (3.51)	17–30	6–30	.72
Consequences	19.34 (5.21)	6–30	6–30	.84
Personal control	17.37 (4.28)	6–27	6–30	.72
Treatment control	15.94 (3.26)	6–23	6–30	.63
Coherence	16.03 (5.30)	5–25	5–25	.92
Timeline (cyclical)	12.05 (3.58)	4–20	4–20	.80
Emotional representations	18.75 (5.64)	7–30	7–30	.90
Psychosocial stressors	14.45 (5.06)	6–28	6–30	.84
External/altered immunity	7.46 (2.53)	4–16	4–20	.75
Risk factor behaviours	3.65 (1.65)	2–8	2–8	.80
Brief cope				
Problem focused	17.61 (6.83)	9–35	9–36	.89
Avoidance focused	8.13 (3.17)	6–24	6–24	.81
Substance use	2.97 (1.56)	2–8	2–8	.87
Humour	3.03 (1.54)	2–8	2–8	.86
Spirituality	3.54 (1.96)	2–8	2–8	.87

The identity subscale is a sum of symptoms associated with CD

### Associations between Clinical and Outcome Variables

Correlations between clinical and demographic variables and study outcomes were performed. A significant relationship between HADS-A and age diagnosed emerged (*r*_s_ = − 0.22, *p* = .018), age (*r*_s_ = − .28, *p* = .002), such that higher anxiety scores were associated with younger current age and age diagnosed. A previous history of mental health difficulty was also associated with higher anxiety scores (*r*_pb_ = .48, *p* < .001). HADS-D (*r*_pb_ = .47, *p* < .001) and the CDIP-58 (*r*_pb_ = .40, *p* < .001)) correlated with mental health difficulty, such that a previous history of mental health difficulties was associated with higher depression scores and poorer HRQoL. PTG scores were negatively correlated with age diagnosed (*r*_s_ = − .29, *p* = .001)) and positively with disease duration (*r*_s_ = .29, *p* = .001), such that younger age of diagnosis and longer time since diagnosis was associated with higher post -traumatic growth.

### Associations between Illness Perceptions, Coping and Outcomes

Several illness perceptions were related to psychological outcomes, as shown in Table 3. A strong illness identity, perception of a cyclical timeline, perception of serious consequences, strong emotional representations and beliefs about psychosocial causes were associated with higher levels of psychological distress, while stronger beliefs in treatment control and coherence were associated with lower distress. A similar pattern was seen for HRQoL, where illness identity, cyclical timeline, consequences and emotional representations were associated with a poorer HRQoL and higher perceptions of personal control, treatment control and coherence were associated with better HRQoL. Problem-focused coping, avoidance-focused coping, spirituality and substance use were also associated with elevated levels of psychological distress and poorer HRQoL, whereas humour was not. Higher PTG was associated with higher illness identity, problem-focused coping and spirituality.

**Table 3:** Correlations between study variables

	1	2	3	4	5	6	7	8	9	10	11	12	13	14	15	16	17	18	19
1. Identity	–																		
2. Timeline (chronic)	− .10	–																	
3. Consequences	.41^**^	.23^*^	–																
4. Personal control	.01	− .19^*^	− .12	–															
5. Treatment control	− .18	− .25^**^	− .31^**^	.26^**^	–														
6. Coherence	− .06	− .01	− .16	.19^*^	.30^**^	–													
7. Timeline (cyclical)	.24^**^	.04	.23^*^	.11	− 0.04	− .25^**^	–												
8. Emotional representations	.42^**^	.06	.53^**^	− .12	− .33^**^	− .44^**^	.30^**^	–											
9. Psychosocial causes	.05	.03	.21^*^	.03	− .08	− .18^*^	.17	.23^*^	–										
10. External/altered immunity	.04	− .21^*^	− .09	− .03	− .14	− .19^*^	.03	.07	.30^**^	–									
11. Risk factors' behaviours	− .25^**^	− .08	− .06	− .04	.06	.01	.07	− .03	.29^**^	.35^**^	–								
12. Problem-focused coping	.27^**^	− .01	.43^**^	.26^**^	.05	.15	.17	.29^**^	− .04	− .13	− .24^**^	–							
13. Avoidance-focused coping	.25^**^	.01	.44^**^	.06	− .12	− .29^**^	.31^**^	.61^**^	.25^**^	− .01	− .07	.40^**^	–						
14. Substance use	.04	.09	.22^*^	− .03	− .10	.00	.04	.25^**^	.23^*^	− .01	.15	.19^*^	.29^**^	–					
15. Humour	− .08	.20^*^	− .06	0.11	− .07	− .08	.09	.02	.04	− .11	.00	.15	.11	.09	–				
16. Spirituality	.09	− .09	.17	0.12	.09	− .05	.20^*^	.22^*^	.10	.13	.18^*^	.33^**^	.12	.06	.13	–			
17. HADS anxiety	.39^**^	.07	.44^**^	− .08	− .25^**^	− .22^*^	.28^**^	.67^**^	.33^**^	− .05	− .03	.38^**^	.60^**^	.38^**^	.17	.20^*^	–		
18. HADS depression	.40^**^	.01	.48^**^	− .14	− .25^**^	− .31^**^	.23^*^	.70^**^	.24^**^	− .03	− .12	.34^**^	.65^**^	.39^**^	− .06	.20^*^	.75^**^	–	
19. PTG	.22^*^	.07	.14	.13	.03	.08	.10	.13	.08	− .02	− .12	.29^**^	.13	.06	− .12	.18^*^	.17	.18	–
20. CDIP-58	.44^**^	.09	.57^**^	− .21^*^	− .34^**^	− .23^*^	.23^*^	.70^**^	.15	.00	− .11	.40^**^	.51^**^	.29^**^	− .01	.27^**^	.72^**^	.73^**^	.27^**^

^*^p < .05, **p < .01

Significant relationships between illness perceptions and coping strategies were also found. Identity, consequences, personal control, emotional representations and risk factor behaviours were associated with increased use of problem-focused coping. Identity, cyclical timeline, consequences, emotional representations and psychosocial causes were associated with the use of avoidance-focused coping, while a stronger illness coherence was associated with reduced avoidance-focused coping. A cyclical timeline, emotional representations and risk factor causes were associated with higher use of spirituality as a coping strategy. Chronic timeline was associated with the use of humour and stronger beliefs in consequences; emotional representations and psychosocial causes were associated with substance use as a coping strategy.

### Hierarchical Multiple Regressions

For HADS Anxiety (Table 4), block 1 (age and history of mental health difficulty) explained 27% (*p* < .001) of variance in anxiety scores. Block 2 (identity, consequences, treatment control, cyclical timeline, emotional representations and psychosocial causes) explained a further 25% (*p* < .001) of variance while block 3 (problem -focused, avoidance-focused and substance use coping strategies) explained a further 7% (*p* < .001) of variance. The overall model explained 59% of variance *F*(11, 105) = 13.44, *p* < .001, adjusted *R*^2^ = 0.54). In the final model, emotional representations (*β* = 0.32), psychosocial causes (*β* = 0.16), problem-focused coping (*β* = 0.20) and substance use coping (*β* = 0.14) had significant and the strongest relationships with HADS Anxiety.

**Table 4:** Final hierarchical multiple regression model for HADS anxiety

	HADS anxiety						
Variable	*b*	SE B	*β*	95% CI	Part	*R*^2^	Δ *R*^2^
*Block 1*						.27	.27
Age	− .03	.03	− .07	[− .09, .03]	− .06		
History of mental health	1.72	.94	.14	[− .14, 3.58	.12		
*Block 2*						.52	.25
Identity	.24	.13	.14	[− .1, .50]	.12		
Consequences	− .08	.09	− .07	[− .26, .11]	− .05		
Treatment control	− .20	.12	− .12	[− .44, .04	− .10		
Cyclical timeline	.06	.11	.04	[− .17, .28]	.03		
Emotional representation	.32	.09	.32***	[.14, .51]	.22		
Psychosocial causes	.19	.08	.16*	[.03, .34]	.15		
*Block 3*						.58	.07
Problem-focused coping	.17	.06	.20**	[.04, .29]	.17		
Avoidance coping	.21	.16	.10	[− .10, .52]	.08		
Substance use	.50	.25	.14*	[.00, .99]	.13		

*p < .05, **p < .01, ***p < .001

For HADS depression scores (Table 5), block 1 (history of mental health difficulty) explained 21% (*p* < .001) of the variance. Block 2, which included the illness perception variables (identity, consequences, treatment control, coherence, emotional representations and psychosocial causes) explained an additional 30% (*p* < .001) of variance in depression score. The addition of problem -focused, avoidance-focused and substance use coping strategies in block 3 explained an additional 11% (*p* < .001) of variance. The overall model explained 61% of the variance in depression scores *F*(10,107) = 16.79, *p* < .001, adjusted *R*^2^ = .57. In the final model, emotional representation (*β* = 0.30*)*, avoidance-focused coping (*β* = 0.27*)* and substance use (*β* = 0.24) were significant and had the strongest relationships with depression.

**Table 5:** Final hierarchical multiple regression model for HADS depression

	HADS depression						
Variable	*b*	SE B	*β*	95% CI	Part	*R*^2^	Δ *R*^2^
*Block 1*						.21	.21
Mental health history	.99	.79	.09	[− .58, 2.56]	.08		
*Block 2*						.51	.30
Identity	.20	.11	.13	[.01, .42]	.11		
Consequences	.04	.08	.05	[− .11, .20]	.04		
Treatment control	− .05	.10	− .04	[− .26, .15]	− .03		
Coherence	− .04	.07	− .04	[− .17, .10]	− .03		
Emotional representations	.26	.08	.30***	[.10, .42]	.20		
Psychosocial causes	.01	.07	.01	[− .12, .14]	.01		
*Block 3*						.61	.11
Problem-focused coping	.04	.05	.06	[− .06, .15]	.05		
Avoidance coping	.41	.12	.27***	[.18, .64]	.21		
Substance use	.76	.21	.24***	[.35, 1.18]	.22		

*p < .05, **p < .01, ***p < .001

For CDIP-58 scores (Table 6), block 1 (history of mental health difficulty) explained 16% (*p* < .001) of the variance. Block 2 , which contained identity, consequences, treatment control and emotional representations, explained an additional 41% (*p* < .001) of variance. Block 3, which contained coping variables of problem focused, avoidance focused, spirituality and substance use, explained an additional 5% (*p* < .001) of variance in health-related quality of life. The overall model explained 61% of the variance in CDIP-58 scores *F*(9,108) = 19.13, *p* < .001, adjusted *R*^2^ = .58. In the final model, treatment control (*β* = − 0.14) and emotional representations (*β* = 0.37) were significant and had the strongest relationship to HRQoL.

**Table 6:** Final hierarchical multiple regression model for CDIP-58

	CDIP-58						
Variable	*b*	SE B	*β*	95% CI	Part	*R*^2^	Δ *R*^2^
*Block 1*						.16	.16
Mental health history	3.67	3.81	.07	[− 3.88, 11.22]	.06		
*Block 2*						.57	.41
Identity	.87	.51	.12	[− .15, 1.89]	.10		
Consequences	.65	.37	.14	[− .07, 1.38]	.12		
Treatment control	− 1.02	.49	− .14*	[− 2.00, -.04]	− .12		
Emotional representations	1.58	.37	.37***	[.84, 2.31]	.25		
*Block 3*						.61	.05
Problem-focused coping	.36	.25	.10	[− .14, .86]	.09		
Avoidance coping	.84	.56	.11	[− .27, 1.94]	.09		
Spirituality	1.22	.78	.10	[− .32, 2.75]	.09		
Substance use	1.83	.99	.12	[− .14, 3.80]	.11		

*p < .05, **p < .01, ***p < .001

For the PTG model, block 1 (age diagnosed and disease duration) explained 8% of the variance in PTG scores while problem-focused coping explained an additional 10% in block 2. The overall model explained 19% of the variance *F*(3, 115) = 8.47, *p* < .001, adjusted *R*^2^ = 16.3%. In the final model (Table 7), both disease duration (*β* = 0.23) and problem-focused coping (*β* = 0.33) were significant.

**Table 7:** Final hierarchical multiple regression model for PTGI

	PTGI score						
Variable	*b*	SE B	*β*	95% CI	Part	*R*^2^	Δ *R*^2^
*Block 1*						.08	.08
Age diagnosed	− .23	.18	− .12	[− .59, .12]	− .11		
Disease duration	.52	.22	.23*	[.08, .96]	.20		
*Block 2*						.19	.10
Problem-focused coping	1.18	.31	.33***	[.56, 1.80]	.32		

*p < .05, **p < .01, ***p < .001

## Discussion

To our knowledge, this is the first study to apply the CSM model to investigate the contribution of illness perceptions and coping strategies to psychological outcomes in pwCD. High levels of psychological distress were found in the current study. Fifty-one per cent of the current cohort experienced symptoms of anxiety, while 28.8% experienced symptoms of depression. These rates are in line with a recent multicentre study which found that 43.3% experienced symptoms of anxiety and 24.5% experienced symptoms of depression (Berman et al., 2017). Furthermore, 27% of the current study indicated that they had a history of difficulties with their mental health, supporting a recent study which found that 23% had a history of clinical diagnosis of mood disorder prior to CD onset and 39% reported having one at any stage (Ndukwe et al., 2020a, 2020b). With regard to HRQoL, the current cohort reported a CDIP-58 score of 35.83, which is substantially lower than a recent multicentre randomised controlled trial (*M* = 59.1; Simonetta-Moreau et al., 2019), which suggests that the current group of pwCD experience a higher HRQoL.

In line with study hypotheses, threat-based beliefs such as a strong illness identity, beliefs in consequences, cyclical timeline, emotional representations and psychosocial causes were consistently related to higher levels of distress and lower HRQoL. These findings are similar to a recent study in MS, where identity, cyclical timeline, emotional representations and psychological causes significantly predicted poorer mental health (Bassi et al., 2020). An interesting finding emerged in relation to timeline beliefs, where stronger relationships between timeline cyclical (beliefs about fluctuating course) as opposed to chronic timeline (beliefs about how long CD will last) were related to poorer psychological outcomes.

In line with the CSM model, it was assumed that chronic timeline beliefs would emerge as a more significant threat-based belief in keeping with the view of CD as a chronic movement disorder with no known cure. A possible explanation for this may be that pwCD view CD as having a cyclical time course, a reflection of the most common treatment and botulinum toxin injections (BTX) at 12–16-week intervals. 94.1% of the participants in the current study were receiving this treatment. The injections provide a respite from motor symptoms during peak efficacy. Symptoms typically re-emerge towards the end of the cycle, which may lead pwCD to view CD as cyclical as opposed to chronic.

Consistent with findings in MS (Bassi et al., 2020), having a coherent understanding of CD was related to better psychological outcomes in the current study, suggesting that making sense of CD, despite its unknown aetiology may be beneficial. Similar to research in PD (Evans & Norman, 2009), the results highlight the positive impact of higher control beliefs on psychological distress and HRQoL, despite individuals having little control over their symptoms. It has been suggested that through accommodating or accepting the health threat, individuals may retain a sense of secondary control, which has been shown to have a positive impact on psychological well-being in MS and PD (Ackroyd et al., 2011; McQuillen et al., 2003).

Attributing CD to psychosocial causes was significantly related to increased distress and poorer HRQoL, a finding which has also been documented in research in MS (Bassi et al., 2020). In the absence of a visible or external cause, as is the case of CD, internal psychological attributions (e.g. my emotional state) can lead to feelings of blame, low self-esteem and low mood (Roesch & Weiner, 2001). Mood and anxiety difficulties precede CD onset in over 50% of pwCD (Conte et al., 2016; Ndukwe et al., 2020a, 2020b), who may be more likely to attribute CD onset to psychological causes/factors or stress (O’Leary et al., 2004). Another possibility is that pwCD may internalise the attributions of healthcare professions: 37% of pwCD were mis-diagnosed with a psychological or stress disorder before receiving a diagnosis of CD (Comella & Bhatia, 2015). Morgan et al. (2019) found that pwCD finds psychological explanations for dystonia stigmatising and a block to seeking psychological support. Given that the aetiology of CD is largely unknown, further research is needed to elucidate and clarify individuals’ causal attributions of CD.

Supporting our second hypothesis, avoidance-focused coping was related to increased distress and lower HRQoL. This is consistent with previous research into PD, which found that cognitive and behavioural avoidance is related to elevated levels of distress (Evans & Norman, 2009; Julien et al., 2016). Generally considered as a maladaptive strategy, avoidance-focused coping may be effective in managing the immediate emotional upheaval associated with a health threat and enhance a sense of control (Hofmann & Hay, 2018). However, prolonged use of avoidance-focused strategies to avoid or escape unpleasant feelings and experiences can maintain and exacerbate distress and is associated with poorer psychological and health outcomes (Hagger & Orbell, 2021).

Our third hypothesis was not supported; problem-focused coping was related to higher levels of distress and to poorer HRQoL. This is in contrast to meta-analytic findings in a range of physical health conditions, where problem-focused coping has been shown to improve psychological distress and HRQoL (Hagger et al., 2017). Generally viewed as an adaptive strategy, it may not be the most effective strategy when faced with an illness that is difficult to control or has limited treatment options such as CD. Problem-focused coping may be perceived as ineffective or as failed attempt to deal with a chronic illness (Roubinov et al., 2015). Our finding is consistent with research in MS and alopecia, where problem-focused coping was associated with increased distress (Cartwright et al., 2009; Roubinov et al., 2015). When there is a strong emotional representation to an illness, as in the current study, emotion-focused strategies, such as venting, which aim to reduce the emotional response as opposed to actively change the source of stress through problem-focused strategies may be more effective (Hagger &Orbell, 2021).

Substance use, humour and spirituality emerged as cohort-specific coping strategies. Both substance use and spirituality were significantly related to psychological outcomes, such that higher use of these strategies was associated with increased distress and lower HRQoL. Humour was not significantly related to any of the psychological outcome variables, which is not surprising given the inconsistent findings in the wider literature as to its efficacy in improving distress and HRQoL (Samson & Gross, 2012). Increases in spirituality or religiousness can imbue hope and meaning in coping chronic illnesses such as MS and PD; however, it can also lead to increased levels of distress in some individuals, as was seen in the current study (Roger & Hatala, 2018). Substance use as a coping strategy was significantly related to anxiety and depression scores. While similar levels of substance use were found in CD relative to the general population, a recent study found that younger males with more severe CD and comorbid depression and anxiety may be at an increased risk of developing substance abuse (Mahajan et al., 2018). Although not formally assessed in the current study, substances may be used as a form of self-medication and given the complex relationships between substance use, psychological and physical health, pwCD should be screened as part of their clinical care (Mahajan et al., 2018). Limited by the cross-sectional design of the study, a causal link between specific coping strategies and psychological outcomes cannot be assumed. The relationships between coping and psychological outcomes are complex and are likely bi-directional. For example, it may be that increased use of a problem-focused coping (or other coping strategies) leads to increased distress or that increased distress leads to more use of problem-focused coping, or the relationship reflects an unmeasured third variable. Longitudinal and experimental research is required to examine the relationships between coping strategies and psychological outcomes for pwCD.

The regression models highlighted the importance of considering the impact of specific illness representations and coping strategies individually across psychological outcomes. Illness perceptions explained 25%, 30% and 41% in anxiety, depression and HRQoL scores, while coping strategies added an additional 7%, 11% and 5% of variance, respectively. After controlling for previous history of mental health difficulty, strong emotional representations emerged as having the strongest relationships to psychological outcomes, suggesting that CD may trigger a strong emotional response in individuals, which may increase distress and HRQoL. This finding is consistent with recent work whereby strong emotional representations were associated with elevated levels of depression in PD (Evans & Norman, 2009) and with poor psychological adjustment in MS (Bassi et al., 2020).

The significant relationships between emotional representations and distress raises the issue of comparable constructs. According to the CSM, emotional representations are viewed as separate to general distress. However, some degree of overlap may be expected and a person’s general level of distress over the last two weeks as measured by the HADS is likely to be influenced by their emotional response to their illness (Dempster et al., 2015).

Beliefs in psychosocial causes and the use of problem-focused coping was significantly related to anxiety. Avoidance-focused coping and substance use coping were also significantly related to depression scores, while higher beliefs in treatment control also accounted for significant amount of variance in HRQoL scores. These findings highlight the significant contributions of illness perceptions and coping strategies to psychological outcomes in pwCD, and psychological interventions addressing both perceptions and coping strategies may be appropriate and helpful in improving psychological outcomes for pwCD.

Post-traumatic growth was also included as an exploratory outcome of psychological well-being, as previous research has demonstrated that it is an applicable construct in other chronic neurological conditions and can have positive effects on physical and mental health (Zeligman et al., 2018). PTG is said to develop out of coping with an adverse experience, such as living with a chronic condition and is typically manifests as changes in self, changes in relationships and changes in philosophy of life (Tedeschi & Calhoun, 1996). PwCD experienced less growth (*M* = 33.62) compared to chronic conditions such as MS (*M* = 43.38) or PD (*M* = 55.53) (Ackroyd et al., 2011; Stutts et al., 2020). This appears in contrast to a study in blepharospasm who reported significantly higher growth scores compared to a hemi-facial spasm clinical control group (Nikolai et al., 2016). It also appears in contrast to a qualitative study in CD that suggested developing new goals, forging a new identity and fostering a positive attitude were important themes for people living with CD (Morgan et al., 2019). Experiencing positive changes as a result of living with a chronic illness is not a universal experience (Barskova & Oesterreich, 2009). Different chronic conditions may experience different types of positive changes, and the use of the PTGI total score as an outcome variable precluded the examination of the proposed five factor structure for pwCD (Purc-Stephenson, 2014). The validity of self-reported PTGI has also been questioned as to whether it represents a genuine positive change for an individual; constructs within the PTGI were not related to actual measures of growth in the general population (Frazier et al., 2009). It may also be that other variables, not measured in the current study such as social support or personality traits are associated with growth (Prati & Pietrantoni, 2009).Exploring other concepts of psychological well-being such as optimism or resilience which can be targeted through psychological interventions may be helpful in the future, for example, higher levels of optimism were related to better well-being PD (Hurt et al., 2014) and muscle disorders (Graham et al., 2014).

### Clinical Implications

Given the high levels of psychological distress in the current study, and reported more widely (Kuyper et al., 2011), the identification of psychological variables such as illness perceptions and coping strategies that can be modified through psychological intervention is encouraging. Illness perception interventions have improved illness outcomes in cardiac, diabetes and psoriasis groups (Petrie & Weiman, 2012). In muscle disorders, a cluster of beneficial illness perceptions, characterised by a realistic timeline, greater coherence, reduced emotional representations and identity, were associated with better HRQoL and improved mood, with authors suggesting that an intervention should aim to address any illness misconceptions and reduce emotional representations (Graham et al., 2013). Although specific to muscle disease, a similar intervention could be explored in CD, given the relationships described in the current study. As a first step, psychoeducation to address illness misconceptions and facilitate the development of beneficial beliefs such as coherence may improve psychological outcomes and improve patient satisfaction (Comella & Bhatia, 2015). The presence of strong emotional representations and high levels of distress indicate that psychoeducation alone may not be sufficient and that pwCD may benefit from psychological interventions to manage the emotional impact of CD (Graham et al., 2013).

Although the evidence for a specific psychological intervention for pwCD is lacking, a recent review of behavioural interventions for AOIFD reported that a combined cognitive behaviour therapy (CBT) and mindfulness-based approach may be beneficial, although cautioned that further high-quality research is needed (Bernstein et al., 2016). Research in other movement disorders, including MS and PD, suggests that CBT can improve psychological well-being and HRQoL and that specific strategies such as relaxation, thought restructuring and behavioural activation may be helpful (Berardelli et al., 2015; British Psychological Society, 2021). Trials for CBT for dystonia are currently underway (Kobayashi et al., 2020; Wadon et al., 2020).

Acceptance and commitment therapy (ACT; Hayes et al., 2006) has been shown to be beneficial in chronic physical health conditions (Brassington et al., 2016) and emerging evidence suggests that it is acceptable, improves QoL and distress in muscle disorders (Graham et al., 2017). In the context of a physical health condition, ACT supports individuals to adapt and develop acceptance of challenges such as pain, disability and loss, associated with their illness by developing greater psychological flexibility rather than trying to avoid or get rid of unwanted experiences (Dindo et al., 2017). ACT may be particularly relevant to CD given the levels of psychological distress, low levels of growth and use of avoidance coping strategies identified in the current sample, which are all indicators of poor adjustment in chronic conditions (Stanton et al., 2007).

### Strengths and Limitations

Due to the cross-sectional design, conclusions regarding causality cannot be made. While illness perceptions and coping strategies accounted for a significant proportion of variance in psychological outcomes, other factors such as self-efficacy, personality and social supports may also have a role (Hagger et al., 2017). Disease status and severity are also key predictors of illness perceptions, which, due to the study design and self-report questionnaires, could not be measured. Validated self-report measures were used to determine the levels of distress; they do not infer the presence of a clinical diagnosis and suggest the presence of self-reported clinically significant symptoms. While a CD-specific measure of HRQoL was used, a generic coping scale was used which does not identify illness-specific coping strategies. In terms of strengths, PCA was applied to examine the higher-order coping strategies instead of adopting a common two- or three-factor model, which can mask cohort-specific strategies (Skinner et al., 2003). The final number of participants with complete datasets in the current study (*n* = 118) was slightly underpowered according to a priori sample size calculation (*n* = 123). However, the number of participants was higher than other studies in neurological conditions applying the CSM (Ackroyd et al., 2011; Hurt et al., 2014), and represented approximately 30% of the CD population of Ireland (Williams et al., 2017). Future research may address these issues by incorporating behavioural beliefs (such as self-efficacy), dispositional traits, an illness-specific coping scale, a measure of disease severity and additional measures of psychological well-being. Future studies could test the CSM longitudinally and examine the proposed mediated relationship of illness perceptions to psychological outcomes through coping strategies (Hagger & Orbell, 2021).

## Conclusions

Illness perceptions and coping strategies are significantly related to distress and HRQoL in pwCD. Psychological interventions are likely to be beneficial in addressing specific beliefs and developing helpful coping strategies to enhance psychological well-being and HRQoL for pwCD. Further research is needed to evaluate the efficacy of specific psychological interventions for pwCD.

## Supplementary Information

Below is the link to the electronic supplementary material.

Supplementary file1 (DOCX 19 kb)

## Data Availability

Contact corresponding author.
