# Supplementary material for: Illness Perceptions, Coping, Health-Related Quality of Life and Psychological Outcomes in Cervical Dystonia
Source: J Clin Psychol Med Settings. 2022 Apr 19;30(1):129–42. doi: 10.1007/s10880-022-09851-2 (PMC10042972; doi:10.1007/s10880-022-09851-2)
Supplement: Supplementary file 1 — Supplementary file1 (DOCX 19 kb) [file 10880_2022_9851_MOESM1_ESM.docx]

Supplementary Table 1

Summary of exploratory factor analysis results for the Brief COPE questionnaire.

| Item | Component | | | | |
| --- | --- | --- | --- | --- | --- |
|  | Problem focused | Avoidant | Spirituality | Humour | Substance use |
| 1. I've been turning to work or other activities to take my mind off things. | **.54** |  |  |  |  |
| 2. I've been concentrating my efforts on doing something about the situation I’m in. | **.77** |  | .31 |  |  |
| 3. I've been saying to myself "this isn't real." |  | **.76** |  |  |  |
| 4. I've been using alcohol or other drugs to make myself feel better. |  |  |  |  | **.89** |
| 5. I've been getting emotional support from others. | **.70** |  |  |  |  |
| 6. I've been giving up trying to deal with it. |  | **.62** |  |  |  |
| 7. I've been taking action to try to make the situation better. | **.65** |  |  |  |  |
| 8. I've been refusing to believe that it has happened. |  | **.70** |  |  |  |
| 9. I've been saying things to let my unpleasant feelings escape. |  | .54 |  |  | .40 |
| 10. I’ve been getting help and advice from other people. | **.77** |  |  |  |  |
| 11. I've been using alcohol or other drugs to help me get through it. |  |  |  |  | **.82** |
| 12. I've been trying to see it in a different light, to make it seem more positive. | .49 |  | .37 |  |  |
| 13. I’ve been criticizing myself. |  | .**72** |  |  |  |
| 14. I've been trying to come up with a strategy about what to do. | **.73** |  |  |  |  |
| 15. been getting comfort and understanding from someone. | **.68** |  |  |  |  |
| 16. I've been giving up the attempt to cope. |  | **.78** |  |  |  |
| 17. I've been looking for something good in what is happening. | .40 |  | .55 |  |  |
| 18. I've been making jokes about it. |  |  |  | **.88** |  |
| 19. I've been doing something to think about it less, such as going to movies, watching tv, reading, daydreaming, sleeping, or shopping. | .48 |  |  | .47 |  |
| 20. I've been accepting the reality of the fact that it has happened. | .38 |  |  |  |  |
| 21. I've been expressing my negative feelings. | .48 | .56 |  |  |  |
| 22. I've been trying to find comfort in my religion or spiritual beliefs. |  |  | **.82** |  |  |
| 23. I’ve been trying to get advice or help from other people about what to do. | **.79** |  |  |  | .33 |
| 24. I've been learning to live with it. | .33 |  |  |  | -.32 |
| 25. I've been thinking hard about what steps to take. | **.75** |  |  |  |  |
| 26. I’ve been blaming myself for things that happened. |  | **.67** |  |  |  |
| 27. I've been praying or meditating |  |  | **.78** |  |  |
| 28. I've been making fun of the situation. |  |  |  | **.87** |  |
| Eigenvalues | 7.37 | 3.25 | 2.14 | 1.92 | 1.70 |
| % of variance | 26.30 | 11.62 | 7.66 | 6.85 | 6.07 |
| α | .89 | .81 | .87 | .86 | .76 |

Note: Factor loadings below .3 have been suppressed. Factor loadings above .5, which were used to form higher order strategies are highlighted in bold.
